# Learn from the Best

**DOI:** 10.1371/journal.pcbi.1003645

**Published:** 2014-05-29

**Authors:** Virginie Bernard, Sebastian J. Schultheiss, Magali Michaut

**Affiliations:** 1 Next Generation Sequencing Platform - ICGex, Curie Institute, Paris, France; 2 Computomics Molecular Data Analysis, Tuebingen, Germany; 3 Computational Cancer Biology, Netherlands Cancer Institute, Amsterdam, Netherlands; Princeton University, United States of America

## Abstract

What is more inspiring than a discussion with the leading scientists in your field? As a student or a young researcher, you have likely been influenced by mentors guiding you in your career and leading you to your current position. Any discussion with or advice from an expert is certainly very helpful for young people. But how often do we have the opportunity to meet experts? Do we make the most out of these situations? Meetings organized for young scientists are a great opportunity not only for the attendees: they are an opportunity for experts to meet bright students and learn from them in return. In this article, we introduce several successful events organized by Regional Student Groups all around the world, bridging the gap between experts and young scientists. We highlight how rewarding it is for all participants: young researchers, experts, and organizers. We then discuss the various benefits and emphasize the importance of organizing and attending such meetings. As a young researcher, seeking mentorship and additional skills training is a crucial step in career development. Keep in mind that one day, you may be an inspiring mentor, too.

## Introduction

Scientists deeply care about mastery of skills in their field of research. In general, we admire people who are extremely good at what they do, and we enjoy doing things we are good at. To improve our skills and our knowledge, it is important to learn the craft from others who are better than us. Scientists defining the field or setting the records and boundaries to be broken by future generations are the mentors one wants to learn from. Getting involved with the right experts is an important undertaking for any student in any field at all levels of training [1].

The Regional Student Groups (RSGs) [2] have focused on promoting the interactions between students and experts in the field of computational biology [3]–[6]. RSGs have been successful in steering students towards a great career [7]. A first step is to provide students with exposure to both (i) experts in scientific disciplines working in relevant fields and (ii) experts excelling in specific skills that one should have or want to have. For example, career advisors, programming teachers, or scientific writing instructors are good experts to meet. Such exposure can be gained through specific presentations tuned to a particular audience, such as students, postdocs, and young researchers.

The prospect to speak to interested students is often enough to pique the interest of a leader in the field. To be able to invite them again, it is necessary to plan and execute the event professionally. We highlight how to achieve that goal in a series of examples throughout the article.

While a university curriculum teaches students how to effectively work in a field, it does not teach them what to do to obtain a position. Most students do not have a clear idea of their career path. The difference between possible job titles, such as research associate, principal investigator, professor, research assistant, or other scientific positions is often not clear to young researchers. They may ask questions about these positions, e.g.: What is the job description? Do I have the skills required? What are the advantages and drawbacks of these positions? Once they know what they want to do, other questions have to be answered. What is the typical journey leading to the position I would like to obtain? Would it help to work abroad to learn from experts in other countries? Knowledge about careers can be gained during seminars or workshops targeted to young researchers. Experts provide feedback on these questions based on their own experience. Students should attend career events to help them find a fitting career, to provide them with guidance on their journey, and to learn soft skills that are not typically taught in university curricula.

RSG activities are beneficial for all students attending them. First of all, a group helps an individual to be bolder. Students that are part of a group—all looking for advice—are in a position to ask more questions. Without supervisors or senior colleagues, students dare to ask almost any question. A question that may seem silly to the person asking it is, in fact, often helpful for the whole group. Student and expert meetings help to extend networks, not only among young scientists but also among organizers and invited experts.

**About the Authors**

The authors have been involved in several aspects of the International Society for Computational Biology (ISCB) Student Council and its Regional Student Groups. **Virginie Bernard** was secretary (2010–2011) of RSG-France and served on its Board of Directors from 2009 till 2012. **Sebastian J. Schultheiss** was president of RSG-Germany (2009–2012). **Magali Michaut** was co-founder and president (2008–2010) of RSG-France and served on its Board of Directors (2008–2013). She was co-founder, secretary (2009), and president (2010–2011) of RSG-Europe and served as secretary for the ISCB Student Council (2009).

### Learn from Various Career Paths and Find Your Own

Since 2009, RSG-France has been organizing an annual one-day, free-of-charge symposium associated with the national conference in bioinformatics. The goal of this symposium is to provide career advice to attendees. Thanks to feedback coming from surveys conducted every year after the symposium, RSG-France takes into account what attendees expect from this meeting. Analysis of these surveys highlights how helpful it is to have advice about careers. Every year, students have a better understanding of what they want to do after this day than before it. Experts with different positions are accessible, and this allows all students to find their own mentor. Due to the success of these events, attendees talk about the symposium with colleagues and friends. They highlight to other Master students, PhD candidates, or young researchers that the talks and discussions with experts helped them with their career path. No better advertisement is possible for RSG-France!

To complement these symposia, RSG-France has been organizing scientific breakfasts. The main goal is to let students focus on specific decisions they will have to make in their careers. For example, the questions, “Working in a private company: with or without a PhD?” or “What about doing research abroad?” were discussed while drinking coffee or tea. With several experts covering each topic, attendees discuss one topic deeply. The organizers have to choose a venue fitting for participants from all around France, each time in a different city. The round table format allows for more discussions and the relatively short duration at the start of the day makes it easier for students to attend as compared to a full-day seminar.

### Learn from the Best Experts in a Specific Field and Improve Your Knowledge

In computational biology, programming is an essential skill. However, formal training is often unavailable to students from a different field. To address this need, RSG-Germany and RSG-Poland organized a three-day workshop focused on the use of the Python programming language. The organizers chose an affordable venue equidistant for all participants. The choice fell on a remote location in the Polish Carpathian Mountains, close to the border to Germany. The event was supported by faculty advisors of both participating RSGs, which helped in financing expenses that occurred before participants paid their fees as well as in attracting Python experts, including the preeminent BioPython developer Peter Cock. Thanks to long-term planning and marketing of the event at different institutions around Germany and Poland, as well as postings to several mailing lists, the workshop had a sufficient number of registered attendees. Beyond developing their skills, students were able to socialize with the invited experts and present their own work during sessions of lightning talks. This event can serve as a model to provide skill-training for computational biology students.

### Learn from the Best Students

During meetings with experts, if there is neither a supervisor nor a professor in the audience, students are less shy. They often feel free to ask many questions, and a more lively debate ensues. In addition, students who have the chance to present their work can get feedback not only from their peers but also from the experts present at the event. Such meetings can lead to new collaborations, bridging the gap between experts and young scientists. Finally, speakers generally enjoy these events quite a lot since they have the chance to interact with highly enthusiastic students and young researchers. Moreover, they know that they have more to bring to them than to a regular audience.

### Organizing a Successful Meeting with Experts

Let's face it, the organization of such events involving experts is not necessarily easy. Many challenges can limit the success of good initiatives. A sufficient amount of time and planning is required. Even when you try to think about everything in advance, new questions and problems arise, so you need to be prepared. In particular, if you want to involve experts in your event, you need to contact them early enough, since they are usually busy.

One of the challenges for young organizers is to have a broad enough vision to know who would be a good expert and would bring something helpful for the attendees. It is essential to have a good match between the expertise of the invited guests and the needs of the audience. The communication skills of the speakers are important: one typically wants a great speaker, able to engage and inspire young scientists and students. Once you have a good idea of which experts you want to invite, be professional in your invitation and be prepared for them to decline. Be persistent when contacting someone. Consider inviting twice; some high-profile experts are likely to miss an email invitation. Do not give contradictory information. You should have a very clear idea about what you want the meeting to be. Communicate this idea with precision to the invited experts so that they know what is expected of them.

### Assess the Event's Success

Fortunately, these challenges also come with numerous rewards for everybody. The presentations are generally inspiring. What is better for a student meeting than having a few talks by renowned experts in the field? It may even be the main reason for some people to sign up for the event, before they realize that the event as a whole is very interesting and rewarding.

Although the organizers do most of the work, they also receive a lot of benefits. In addition to what we described above, the organizers (i) expand their network by working together and contacting the experts, (ii) develop soft skills (organization, communication, leadership) essential in research but unfortunately not often taught, and (iii) have a lot of fun preparing and running the event. In the end, the best reward for all this hard work is the satisfaction of the attendees and their willingness to recommend the event. Only then will the continuity of such an event be guaranteed.

Do not hesitate to organize meetings with experts and students: they are all enthusiastic and you will be rewarded. Once you have a good idea of a topic, form a group to organize the meeting, be sure to have a great set of experts, and enjoy the meeting, seeing the benefits for all—including yourself!
